# Effect of levetiracetam and valproate on late-onset post-traumatic seizures

**DOI:** 10.1186/s42494-023-00121-8

**Published:** 2023-04-23

**Authors:** Yanli Wang, Yiqi Wang, Huifang Wang, Xiaoping Du, Jie Miao, James X. Tao, Meizhen Sun

**Affiliations:** 1 Department of Rehabilitation, Shanxi Traditional Chinese Medical Hospital, Taiyuan, 030012 China; 2 Department of Neurology, The First Hospital of Shanxi Medical University, Taiyuan, 030012 China; 3 Department of Neurology, University of Chicago, Chicago, IL 60601 USA

**Keywords:** Levetiracetam, Valproate, Post-traumatic seizure

## Abstract

**Background:**

To compare the preventive effects of levetiracetam and valproate on late-onset post-traumatic seizures in patients with traumatic brain injury (TBI).

**Methods:**

A total of 95 patients with TBI were recruited from 2017 to 2020. They were randomized into three groups: levetiracetam (LEV) group (*n* = 30) receiving LEV treatment (500 mg, bid, po); valproate group (*n* = 32) receiving sodium valproate (500 mg/d, once daily, po); and control group (*n* = 33) receiving no anti-seizure medication. LEV and valproate were given to corresponding groups within seven days after TBI, and the administration lasted for one month. The incidence of epilepsy and adverse events were evaluated at 7 days and 12 months post-TBI.

**Results:**

The cumulative incidences of late post-traumatic seizures at the 12-month follow-up in the LEV, valproate, and control groups were 3.33%, 12.50% and 15.63%, respectively. The cumulative incidence of late post-traumatic seizures in the LEV group was significantly lower than those in the valproate and control groups (*P* < 0.05). The cumulative incidence of late post-traumatic seizure in the valproate group was not significantly different from that in the control group (*P* > 0.05).

**Conclusions:**

LEV can reduce the cumulative incidence of late post-traumatic seizures, whereas valproate can not.

## Background

With increased incidence of traffic accidents, traumatic brain injury (TBI) has become a common and frequently occurring condition, and one of the most common etiologies leading to secondary epilepsy [1, 2]. The incidence of post-traumatic seizures (PTS) after severe TBI ranges 10–20% [3]. Two types of PTS have been identified: early post-traumatic seizures (EPTS) and late post-traumatic seizures (LPTS). EPTS occur within one week post-TBI, whereas LPTS usually occur over one week after TBI [4]. Focal epilepsy and generalized epilepsy are more common after cerebral trauma, while mixed epilepsy, psychomotor epilepsy or non-convulsive epilepsy are less common.

LPTS is one of the most medication-resistant epilepsies and has serious impacts on the physical and mental health of patients with TBI [5]. Risk factors for PTS include old age, penetrating injury, and a higher degree of injury (eg, an intracranial hemorrhage, a midline displacement greater than 5 mm, and a longer duration of coma). Several clinical trials have established that traditional anti-seizure medications (ASMs) can prevent EPTS but not LPTS [6-8]. Valproate acid (VPA) is a commonly used medication for epileptic seizures, but its administration is associated with neurotoxic effects. In contrast, no such adverse effects have been reported for levetiracetam (LEV) treatment. In addition, clinical studies have demonstrated favorable side effects and pharmacokinetic data, as well as high efficacy of LEV. LEV is a broad-spectrum ASM that is widely used for the treatment of partial and generalized epilepsy. However, whether LEV can prevent or reduce LPTS remains unclear [9-12]. VPA and phenytoin are commonly used to prevent PTS in neurosurgery departments in China. In an earlier investigation, the rates of LPTS did not differ significantly between the VPA and phenytoin treatment groups (*P* > 0.05) [13]. In this study, we aimed to evaluate LEV prevention of LPTS in comparison to the effect of VPA.

## Methods

### Subjects

This study was approved by the Institutional Review Board at the First Hospital of Shanxi Medical University, and informed consent was obtained from all patients. Ninety-five patients with TBI who were admitted in the Neurosurgery and Emergency Departments of the First Hospital of Shanxi Medical University were included in this study (32 females, 63 males; mean age 44.6 ± 2.17 years, range 20–65 years). The inclusion criteria were: (1) age of 18–70 years; (2) seizures within 30 min after TBI with at least one of the following conditions detected by CT: brain contusion, subdural hematoma, epidural hematoma, and intracranial hematoma. The exclusion criteria were as follows: (1) a family history of epilepsy; (2) patients with epilepsy before TBI; (3) pregnancy and lactation; (4) patients who did not receive ASM treatment within seven days after TBI; (5) patients with any of the following medical conditions: hypertension, diabetes, stroke, renal and hepatic dysfunctions, and malignant tumor.

Of the 95 patients, 25 had simple brain contusion (SBC), 27 had SBC and subdural hematoma, 18 had SBC and epidural hematoma, and 25 had SBC and multiple intracranial hematomas. Glasgow coma score (GCS) [14] was assessed within 4 h post-TBI (Table 1). There were 16 severe (GCS 3–7), 33 moderate (GCS 8–12), and 46 mild cases (GCS 13–15).

**Table 1:** Demographics and clinical data of the patients with acute brain injury

	***n***	**Sex (M/F)**	**Age (mean ± SD)**	**GCS score (mean ± SD)**	**EPTS** ***n***	**Brain injury**			
						**SBC**	**SBH**	**EPH**	**MIH**
LEV group	30	20/10	40.9 ± 2.03	12.1 ± 2.10	3	10	9	5	6
VPA group	32	23/9	45.2 ± 1.81	11.9 ± 1.91	2	7	8	9	8
Control group	33	20/13	44.2 ± 3.22	10.4 ± 1.75	3	8	10	4	11
*P*			0.56	0.87	> 0.05	> 0.05			

*Abbreviations: EPTS* early post-traumatic seizures, *GCS score* The Glass Coma Scale score, *SBC* simple brain contusion, *SBH* Subdural hematoma, *EPH* epidural hematoma, *MIH* multiple intracranial hematomas

### Pharmacological treatment with ASMs

The 95 patients were randomized into three groups by a random number table: (1) the LEV group, in which they were treated with 500 mg LEV twice daily after brain injury, for one month; (2) the VPA group, in which they were treated with 500 mg VPA once daily after brain injury, for one month; and (3) the control group, in which they received no ASM treatment. In the VPA group, although the minimum dose of sodium valproate was recommended to be 600 mg/day, it was administered at 500 mg/day for convenience as the sodium valproate sustained-release tablets were 500 mg each. If the patients could not take drugs orally, the medication was given through a nasogastric tube. The medication was started at an average of 4.5 days after brain injury.

The seizure frequency of LPTS was recorded for 12 months after brain injury.

### Statistical analysis

Data of age, GCS, and the number of diagnoses were analyzed with One-way ANOVA test. Pearson χ^2^ test was applied to compare the EPTS, brain injuries, and the cumulative incidence of LPTS among groups at 12 months. All analyses were made using the SPSS 17.0 software. *P* < 0.05 was considered as statistically significant.

## Results

### Patient follow-up

All patients in the LEV and VPA groups completed the12-month follow-up. One patient in the control group was lost to follow-up due to the wrong telephone number provided. Both LEV and VPA were well tolerated by the patients. One patient experienced brief dizziness during the LEV treatment, but it was improved a few days later. One patient experienced mild insomnia during the VPA treatment, which diminished three days later. Another patient in the VPA group had mild gastrointestinal discomfort that improved one day later.

### Cumulative incidence of seizures

The incidence of LPTS was significantly higher in the LEV group than in the VPA group (3.33% vs 12.50%, *χ*^2^ = 4.41, *P* = 0.031) and control group (3.33% vs 15.63%, *χ*^2^ = 6.07,* P* = 0.017). There was no statistically significant difference in the incidence of LPTS between VPA and control groups (*χ*^2^ = 1.15, *P* = 0.36).

These findings indicated that LEV had a significant preventive effect against LPTS, whereas VPA did not (Table 2).

**Table 2:** Comparison of the cumulative incidences of LPTS in different groups

Group	*n*	Lost to follow up (*n*)	*n* of patients with seizure attacks	Cumulative incidence (%)
LEV	30	0	1	3.33
VPA	32	0	4	12.50*
Control	33	1	5	15.63*

^*^: Compared with the LEV group, *P < 0.05

## Discussion

In this study, we found that the cumulative incidence of LPTS in the LEV group was significantly lower than those in the VPA and control groups. The incidence of LPTS in the VPA group was not significantly different from that in the control group. These findings indicated that LEV could prevent LPTS in patients suffering from TBI, whereas VPA could not.

Our results were consistent with those of Pearl et al. published in 2013 [10]. In their study, LEV (55 mg/kg per day bid for 30 days, starting within 8 hours post-brain injury) more effectively prevented LPTS in 20 TBI children aged 6–17 years with high risk factors of developing posttraumatic epilepsy. They found that only 1 of the 20 patients developed LPTS during the two-year follow-up period.

With respect to adverse events, brief dizziness occurred in the LEV group, which disappeared a few days later. Pearl et al. found that the most common severe adverse events were headache, fatigue, drowsiness and irritability, and there was a higher incidence of infection, mood change, or behavior problems among the treatment patients compared to the observation patients. This may be because that the dose of LEV (adult, 1000 mg/day) was better tolerated in the adult patients in our trial than in the pediatric patients (55 mg/kg per day) in the Pearl’ trial.

Our result was also consistent with the the study of Klein et al. in 2012 [9]. In their study, treatment with LEV at 55 mg/kg per day lasted for 30 days, starting within 8 hours post-brain injury. They found that 15.1% (13/86) of the total adults developed epilepsy two years post-brain injury. More specifically, 10.9% (5/46) of the treated adults and 20% (8/40) of the untreated adults developed epilepsy two years post-brain injury (relative risk, 0.47; *P* = 0.18). However, although LEV tended to prevent LPTS, this effect was not statistically significant [9].

In the trial by Klein et al., LEV treatment was terminated in 3% of the patients in the LEV group due to somnolence. The most common adverse events were fatigue, headache, and somnolence. The mood scores and number of infections did not differ between the LEV group and the control group [9]. In another study, LEV treatment at 2000 mg/day caused somnolence, manifested as reduced awake time during the Maintenance of Wakefulness Test and increased nap duration and episodes [15]. However, a daily dose of 1000 mg caused no changes in the daytime sleepiness in the Multiple Sleep Latency Test [16]. Therefore, LEV dosage of 1000 mg/day was used in our trial for the prevention of LPTS without somnolence. In studies of Pearl et al. [10] and Klein et al. [9], LEV was administered at the dose of 55 mg/kg per day, and both studies reported common adverse events of headache, fatigue, and drowsiness, indicating that the dose of LEV for prevention of LPTS needs to be reduced (< 55 mg/kg per day) in clinical settings [9, 10].

Our results were also consistent with the study conducted by Chaudhry et al. [17], which reported that LEV caused less adverse effects and had a better tolerability. Although the pathogenesis of LPTS is not fully clear, this condition can be prevented during its incubation period after TBI [13].

Laure Peter-Derex et al. conducted a phase 3 PEACH trial in France, in which patients who had a non-traumatic intracerebral hemorrhage received LEV treatment, starting within 24 h after hemorrhage. They found that LEV effectively prevented acute seizures in intracerebral hemorrhage [18].

There are several limitations in this study. First, the blood concentration of LEV and VPA were not measured in the patients. Second, the study was not blinded, which might have caused a bias in analysis. Third, the sample size was relatively small. Fourth, the duration of follow-up was relatively short. Multicenter, randomized, placebo-controlled trials are needed in the future to explore the preventive effects of LEV against LPTS.

## Conclusions

In this study, we found that VPA could not prevent LPTS and caused more adverse events. This is consistent with the findings of Temkin et al. and Paqnl et al. [13, 19]. Valproate sodium can exert antiepileptogenic effects at high doses that may be toxic for human use. However, LEV can effectively reduce the cumulative incidence of LPTS in patients with TBI.

## Data Availability

Not applicable.

## Abbreviations

ASM: Anti-seizure medication
EPTS: Early posttraumatic seizure
GCS: Glasgow coma scale
LEV: Levetiracetam
LPTS: Late posttraumatic seizure
PTS: Post-traumatic seizures
SBC: Simple brain contusion
TBI: Traumatic brain injury
VPA: Valproic acid
